# The Impact of Divergent Algal Hydrocolloids Addition on the Physicochemical, Viscoelastic, Textural, and Organoleptic Properties of Cream Cheese Products

**DOI:** 10.3390/foods12081602

**Published:** 2023-04-10

**Authors:** Anna Vincová, Kristýna Šantová, Vendula Kůrová, Alena Kratochvílová, Veronika Halámková, Markéta Suchánková, Eva Lorencová, Daniela Sumczynski, Richardos Nikolaos Salek

**Affiliations:** 1 Department of Food Technology, Faculty of Technology, Tomas Bata University in Zlin, nám. T. G. Masaryka 5555, 760 01 Zlin, Czech Republic; a_vincova@utb.cz (A.V.); k_santova@utb.cz (K.Š.); v_kurova@utb.cz (V.K.); a_jedounkova@utb.cz (A.K.); v_halamkova@utb.cz (V.H.); m_suchankova@utb.cz (M.S.); lorencova@utb.cz (E.L.); 2 Department of Food Analysis and Chemistry, Faculty of Technology, Tomas Bata University in Zlin, nám. T. G. Masaryka 5555, 760 01 Zlin, Czech Republic; sumczynski@utb.cz

**Keywords:** cream cheese, hydrocolloids, viscoelastic properties, κ-carrageenan, furcellaran, ι-carrageenan, sodium alginate

## Abstract

The aim of the current study was to evaluate the addition of different algal hydrocolloids (κ-carrageenan, ι-carrageenan, furcellaran, and sodium alginate) at three different concentrations (0.50, 0.75, and 1.00% *w*/*w*) on the physicochemical, viscoelastic, textural, and organoleptic properties of model cream cheese (CC) samples. On the whole, the highest viscoelastic moduli and hardness values of the CC samples were reported when κ-carrageenan was used. Furthermore, increasing the concentrations of the tested hydrocolloids led to increases in the viscoelastic moduli and hardness values of CC. Recommendations for softer-consistency CC production include the application of κ-carrageenan at a concentration of 0.50–0.75% (*w*/*w*) or the use of furcellaran and sodium alginate at a concentration of 1.00% (*w*/*w*). For the production of CC with a more rigid consistency, it is recommended to apply κ-carrageenan at a concentration higher than 0.75% (*w*/*w*).

## 1. Introduction

In general, cream cheese (CC) can be described as a soft, fresh, and acid curd dairy product that belongs to the unripened cheeses. In addition, CCs are oil-in-water emulsions manufactured by acidification with lactic acid bacteria and heat treatment, together with mechanical stress. The manufacture of CC is based on the slow-rate gelation (induced by acids) of homogenized and thermally treated milk and cream mixtures in the presence of mesophilic cultures, optionally supported by the addition of cheese-making coagulants (rennets), and the subsequent separation of the whey [1,2]. CCs are popular for their mild to slightly sour flavor and their most important characteristics are their smooth and spreadable consistency and creamy mouthfeel. The popularity of CC is also attributed to its versatile use in the food industry, e.g., as ingredients in baked goods, desserts, and salads, or as a raw material in spreads or cheesecakes [3,4]. Furthermore, the hardness and spreadability are fundamental properties of CC and are influenced by several factors [5], including manufacturing parameters (temperature during manufacturing, stirring speed, and overall manufacture time) and the possible application of hydrocolloids as texture modifiers/enhancers [6,7].

Hydrocolloids belong to a large heterogeneous group of hydrophilic biopolymers and substances of high-molecular-weight polysaccharide or protein natures [8]. Hydrocolloids for CC production are mainly used as stabilizers or thickeners. In general, the reasons for the use of hydrocolloids are (i) their ability to bind water, thus directly affecting the rheological properties of the product, (ii) their ability to modify the flow behavior or viscosity, as well as modify the textural properties, and, in particular, (iii) to prevent water release during prolonged storage [9]. Hydrocolloids extracted from red seaweed *Rhodophyceae* are called carrageenans. Carrageenans (also known as ion-sensitive biopolymers) are linear polysaccharides that contain the structural unit D-galactopyranose. Their basic structure is formed by repeating sequences of β-D-galactopyranose and 3,6-anhydro-α-D-galactopyranose (DA units) in a disaccharide called carabiose. In the food industry, attention is paid mainly to the three most significant carrageenan fractions, namely κ-, ι-, and λ-carrageenan, in which the DA units and sulfate residues are different. Kappa-carrageenan has one sulfate ester per galactose dimer, ι-carrageenan has two sulfate esters per galactose dimer, and λ-carrageenan has three sulfate esters [10,11,12]. Another difference between these fractions is that κ-carrageenan and ι-carrageenan are capable of forming gels, but λ-carrageenan does not form gels, is highly soluble, and is only used as a thickener [13]. Furthermore, furcellaran is a sulfated polysaccharide naturally occurring in the algae species *Furcellaria lumbricalis*. Furcellaran is composed of galactose and 3,6-anhydrogalactose esters with copolymers of sodium, potassium, calcium, magnesium, or ammonia. The hexoses are alternately linked by α-(1,3) and β-(1,4) bonds. Furcellaran resembles κ-carrageenan in its properties and structure, and is, therefore, generally classified as a carrageenan, but the difference lies in the number of ester-linked sulfate groups present [14]. Furcellaran possesses one sulfate group per tetramer at position four of the galactose unit; however, κ-carrageenan contains one sulfate group per galactose dimer [15]. The term alginate describes the salts of alginic acid, which are sodium, potassium, ammonium, and calcium, that predominantly occur in brown seaweeds. However, only sodium alginate is commonly used in the food industry [16]. Alginates form linear, covalently linked polymers composed of D-mannuronic acid and L-glucuronic acid. These polysaccharides are highly hydrophilic and are used in the dairy industry as thickening agents or stabilizers [17].

In general, there is little information regarding the effect of different algal hydrocolloid addition on the consistency (a property described mainly based on rheological and textural parameters) of CC. In particular, information on the effect of furcellaran on selected properties of CC is scarce in the literature, although κ- and ι-carrageenans have been described in detail. In addition, the use of furcellaran or sodium alginate in CC products is rare, and no information on their application in CC manufacture is available in the scientific literature. The present study was undertaken with the main objective of examining the impacts of κ-carrageenan (KC), furcellaran (FR), ι-carrageenan (IK), and sodium alginate (AS) (in concentrations of 0.50, 0.75, and 1.00% *w*/*w*) addition to CC and evaluate their physicochemical, viscoelastic, textural, color, and organoleptic properties.

## 2. Materials and Methods

### 2.1. Raw Materials Used for the Manufacture of the Cream Cheese Samples

The following raw materials were used in the manufacture of CC: quark cheese (Choceňská mlékárna s.r.o., Choceň, Czech Republic) with a dry matter (DM) content of 27% (*w*/*w*) and a fat in dry matter (FDM) content of 11% (*w*/*w*), sour cream (Bohemilk, a.s., Opočno, Czech Republic) with a DM content of 36% (*w*/*w*) and a FDM content of 40% (*w/w*), water, NaCl, and κ-carrageenan (Cas Number: 11114-20-8; molecular mass: 4.31 × 10^5^ Da; SigmaAldrich, Ltd., Prague, Czech Republic), ι-carrageenan (CAS Number: 9005-38-3; molecular mass: 7.88 × 10 Da; SigmaAldrich, Ltd., Prague, Czech Republic), sodium alginate (CAS Number: 9005-38-3; molecular mass: 2.16 × 10 Da; SigmaAldrich, Ltd., Prague, Czech Republic), or furcellaran (CAS Number: 9000-21-9; molecular mass: 2.55 × 10^5^ Da; Est-agar, Estonia).

### 2.2. Manufacture of the Cream Cheese Samples

The raw material composition (Table 1) of the model samples was designed to achieve the final CC products with a DM content of 27% (*w*/*w*) and a FDM content of 33% (*w*/*w*). The quark cheese was first mechanically ground on the processing equipment (Vorkwerk Thermomix TM; Vorwerk & Co. Thermomix GmbH, Wuppertal, Germany) and subsequently mixed with the sour cream. In the next step, the remaining raw materials (NaCl, hydrocolloids, and water) were added and mechanical stirring was carried out at 3000 rpm in the presence of heat. The target processing temperature was set at 80 °C, with a holding time of 10 min. The total processing time was 15 min. Hydrocolloids were added separately at concentrations of 0.50, 0.75, and 1.00% (*w*/*w*). Furthermore, a control sample (CS) was manufactured without hydrocolloid addition. The hot mass was then poured into 100 g laminated aluminum containers (conical shape; inner dimensions of 26.8 mm height, 81.1 mm diameter at the top, and 68.9 mm diameter at the bottom) and sealed with aluminum lids using the NovaSeal sealing equipment (Nirosta Ltd., Chlumec nad Cidlinou, Czech Republic). The CC sample weight in one packaging container was approximately 87 ± 5 g. The samples were left to cool and stored at 6 ± 2 °C until subsequent analyses were performed. All analyses were conducted after 7 days of storage (at 6 ± 2 °C). The experiment was carried out 3 times, and a total number of 39 samples (*n* = 39) were manufactured.

### 2.3. Basic Physicochemical Analysis of the Cream Cheese Samples

The DM and fat contents were determined according to ISO 5534:2004 [18] and ISO 1735:2004 [19], respectively. pH was determined using a pH meter equipped with a glass-tipped electrode (Foodcare HI 99161, Hanna Instruments Czech s.r.o., Prague, Czech Republic) in the CC samples at 3 randomly selected locations. The AquaLab 4TE apparatus (Qi Analytical, s.r.o., Prague, Czech Republic, Decagon) was implemented for the determination of the water activity (*a_w_*) of the CC samples at 25.0 ± 0.1 °C. A standard solution (*a_w_* = 0.92 NaCl 2.33 molar in H_2_O; Qi Analytical, s.r.o., Prague, Czech Republic) was included before and during measurement in order to verify the results’ precision. The analyses were performed 9 times (3 manufactured batches × 3 repetitions; *n* = 9).

### 2.4. Rheological Analysis of the Cream Cheese Samples

The determination of the CC’s viscoelastic properties was carried out using a dynamic oscillatory shear rheometer (Thermo Scientific^TM^ RheoStress 1; HAAKE Bremen, Germany) equipped with a parallel plate geometry (with a diameter of 35 mm), and a gap of 1 mm was employed. During the frequency sweeps (in the range of 0.1–100.0 Hz), the shear stress amplitude was set at 20 Pa and the whole measurement was performed within the linear viscoelasticity region. The viscoelastic properties were measured at least 9 times for each model CC sample and the elastic modulus (*G*′) and viscous modulus (*G*″) were recorded during the analysis. From these parameters, the complex modulus of elasticity (G*) was calculated using the following Equation (1):

(1) $$G^{*}=\sqrt{{\left(G^{\prime }\right)}^{2}+{\left(G^{\prime \prime }\right)}^{2}}$$

Moreover, Winter’s critical gel theory was implemented for a more accurate evaluation of the viscoelastic properties in the developed CC samples. Hence, the G* was expressed according to the following Equation (2):

(2) $$G^{*}\left(f\right)=A_{F}\cdot f^{\frac{1}{q}}$$

where *A_F_* (Pa·s^1/z^) is the gel strength, *f* represents the frequency (Hz), and *q* corresponds to the interaction factor, which is defined as the number of structural units interacting in a three-dimensional network [20,21].

### 2.5. Texture Profile Analysis and Spreadability Determination of the Cream Cheese Samples

The determination of the textural properties and spreadability of the CC was performed using a TA.XT plus texture analyzer (Stable Micro Systems Ltd., Godalming, UK). A texture profile analysis was performed on the samples (at 20 ± 1 °C) to ensure 25% deformation by a 20 mm-diameter cylindrical probe (P20); the rate of penetration was 2 mm·s^−1^ and the trigger force was 5 g. The CC container (conical shape; inner dimensions of 26.8 mm height, 81.1 mm diameter at the top, and 68.9 mm diameter at the bottom; and sample weight of approximately 87 ± 5 g) was positioned under the probe and further compressed. From the recorded force–time curves, the textural attributes of hardness, cohesiveness, and gumminess were evaluated [22]. Spreadability was determined with a cone-shaped main probe (male; 90 °) and Plexiglas cone-shaped analyte holders (female). CC samples were applied to the lower cone (female) and the excess sample was gently removed. The tested samples were subsequently penetrated by the upper cone at a 45° angle. The penetration rate into the sample was 1.0 mm/s at a depth of 2.0 mm. During measurement, the sample tended to flow out at a 45° angle, and the degree of spreadability was due to the ease of flow. The removal of the cone probe from the sample provided information on the adhesive properties of the product being analyzed. In both texture profile analysis and spreadability determination, for each attribute examined, the average of a minimum of 3 samples of CC were used for the statistical analysis (3 manufactured batches × 3 repetitions; *n* = 9).

### 2.6. Instrumental Determination of the Color and Emulsion Stability of the Cream Cheese Samples

The instrumental color determination of the CC samples was performed with an UltraScan PRO spectrophotometer (Hunter Associates Laboratory, Inc., Reston, VA, USA). The CIE Lab color scale (L*a*b*) was used for the evaluation with the illuminant D65 (standard daylight) and the 10° angle. The reflectance mode was set for the calibration of the spectrophotometer, with specular reflection excluded, using white (A41 1014-635 Rev. B; Hunterlab ColorFlex CZ; Hunter Associates Laboratory, Inc.) and black (A41-1017-037 Rev A; Hunterlab ColorFlex CZ; Hunter Associates Laboratory, Inc.) reference tiles. Additionally, the parameter L*, reflecting lightness (brightness), corresponded to values in the range of 0–100 (0—black, 100—white). Parameter a* denoted the red to green spectrum (from green – a* to red + a*) and parameter b* denoted the yellow to blue spectrum (from blue – b* to yellow + b*) [23]. The hue angle (h* °) specified the degree of the dominant spectral component, such as red, green, and blue, ranging from 0 ° to 360 °. In particular, an angle of 0° or 360° indicated a red hue, whereas angles of 90°, 180°, and 270° indicated yellow, green, and blue hues, respectively. In general, the combination of a*, b*, and h* ° describes color in a more detailed manner; it is calculated based on the following Equation (3):

(3) $${}^{\circ}h={tan}^{-1}(a^{*}/b^{*})$$

The chroma (C*) represents the saturation of a color, and it was defined as follows (4):

(4) $$C^{*}={\left(a^{*2}+b^{*2}\right)}^{0.5}$$

Moreover, the whiteness index (*WI*) of the CC samples was calculated according to the following formula (5):

(5) $$WI={\left[{\left(100-L^{*}\right)}^{2}+a^{*2}+b^{*2}\right]}^{0.5}$$

Color changes were also evaluated as the total color difference (*∆E12*), indicating the magnitude of color difference between any two samples according to the following formula (6):

(6) $${∆Ε12}^{*}={\left[{\left(∆L^{*}\right)}^{2}+{\left(∆a^{*}\right)}^{2}+{\left(∆b^{*}\right)}^{2}\right]}^{0.5}$$

The determination of the emulsion stability (*S*) of the CC samples was performed according to the study by Nikzade et al. [24], in which 5 g (*m1*) of the CC sample was placed in a polypropylene centrifuge tube (50 mL in volume; 29.1 mm inner diameter, 114.4 mm height, and conical bottom), and the tube was then sealed with a plastic cap. Samples were centrifuged at 6000 rpm for 30 min in a centrifuge (EBA 21 Hettich Zentrifugen, Huttlingen, Germany). Subsequently, the supernatant was thoroughly removed and the resulting sediment was weighed (*m2*). *S* was determined from the following Equation (7):

(7) $$S=\frac{m2}{m1}\cdot 100$$

where *m1* (g) is the mass of the CC model sample placed in the tube and *m2* (g) is the mass of the sediment after draining excess liquid. All CC samples were measured at least 9 times (3 manufactured batches × 3 repetitions; *n* = 9).

### 2.7. Sensory Analysis

A total of 12 assessors (8 women and 4 men from 24 to 52 years old) trained according to ISO 8586 [25] participated in the sensory analysis of the model CC samples. The assessors tested the organoleptic properties of the CC, which were presented to the assessors sequentially, in random order, on white plates and labeled with four-digit codes. Water and crackers were provided to rinse the mouth between the evaluation of the tested CC samples to avoid carryover effects. Sensory analysis was performed in sensory booths under normal lighting conditions according to ISO 8589 [26] and at a controlled temperature (22 ± 2 °C). Furthermore, the CC samples were evaluated using a 7-point intensity scale. For this study, a total of 6 organoleptic attributes (appearance, consistency, flavor, hardness, spreadability, and off-flavor) were evaluated. The following scales were used for the assessment of the model CC samples: a 7-point scale (1–excellent, 4–good, and 7–unacceptable) for appearance, consistency, flavor, and spreadability; a 7-point scale (1–soft, 4–medium, and 7–extra hard) for hardness; and a 7-point scale (1–negligible, 4–medium, and 7–excessive) for off-flavor. Terms for intensity scales were used to describe each point of the applied 7-point scales.

### 2.8. Statistical Analysis

The normal distribution (Shapiro–Wilk test; significance level of 0.05; Minitab^®^ 16 software; Minitab Ltd.; Coventry, UK) of the obtained results was tested. In particular, the application of parametric tests was denied as the normal distribution was not acceptable for all results (*p* < 0.05). Furthermore, the results obtained were processed using the non-parametric analysis of variance through the Kruskal–Wallis and Wilcoxon tests (Minitab^®^ 16 software; Minitab Ltd.; Coventry, UK), with the significance level set at 0.05. The effects of hydrocolloid type and hydrocolloid concentration addition were evaluated separately.

## 3. Results and Discussion

### 3.1. Basic Physicochemical Analysis of the Cream Cheese Samples

The basic physicochemical analysis results of the CC samples are shown in Table 2. The DM content values for all model CC samples ranged from 29.27–29.76% (*w*/*w*) (*p* ≥ 0.05). The similarity of the DM content among the samples is quite crucial, as this factor can significantly affect their textural and rheological properties [27]. The pH value is another factor that affects the textural and rheological properties of the product [28]. Table 2 shows the resulting pH values of CC. Thus, regardless of the type and concentration of hydrocolloid used, the pH values ranged from 4.18–4.23, and it can be assumed that the addition of hydrocolloids did not have a significant effect on the pH values (*p* ≥ 0.05). The resulting pH values could be characterized as acceptable for CC [29,30]. In the case of the determination of *a_w_*, the resulting values (Table 2) were in the range of 0.9817–0.9982 (*p* ≥ 0.05). In the work by Glass and Doyle [11], it was reported that the optimal *a_w_* values for some dairy products were in the range of 0.91–0.96. Furthermore, within the values of the latter interval, the growth of some microorganisms was inhibited. In our study, the resulting *a_w_* values agreed with the results of Møller et al. [31]. According to the authors mentioned above, CC is considered a safe food product due to its physicochemical properties, production technology, and suitable storage conditions.

### 3.2. Rheological Analysis of the Cream Cheese Samples

**Figure 1:**
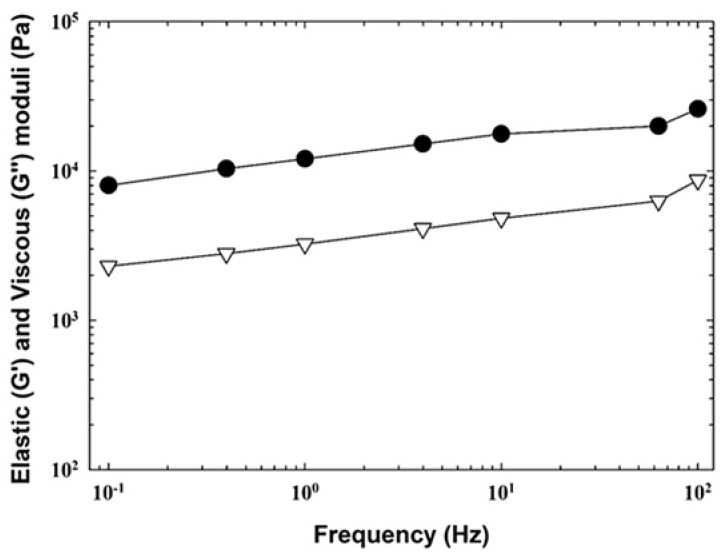
Elastic modulus (*G*′; closed circle; Pa) and viscous modulus (*G*″; open triangle; Pa) development of the control cream cheese sample manufactured without hydrocolloid addition by frequency (ranging from 0.1 to 100.0 Hz) after 7 days of storage (6 ± 2 °C; *n* = 3).

**Figure 2:**
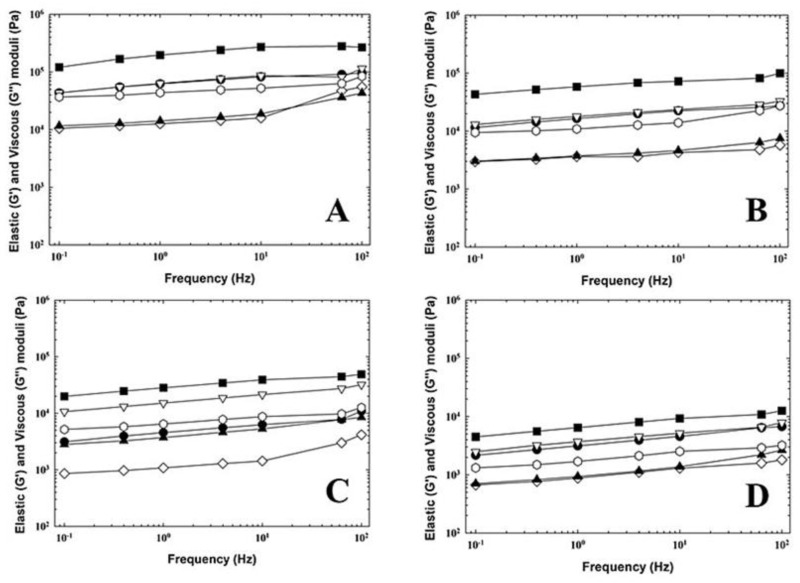
Effect of hydrocolloid concentration on the elastic modulus (*G*′; closed symbols; Pa) and viscous modulus (*G*″; open symbols; Pa) development of the model cream cheese samples manufactured with κ-carrageenan (Part (**A**)), furcellaran (Part (**B**)), ι-carrageenan (Part (**C**)), and sodium alginate (Part (**D**)) in concentrations of 0.50 (triangle), 0.750 (circle), and 1.00 % (square) *w*/*w* by frequency (ranging from 0.1 to 100.0 Hz) after 7 days of storage (6 ± 2 °C; *n* = 3).

Rheological analysis is a key method for the food industry, as it can provide information on the mechanical properties of foods, and the consistency of the product being analyzed can be evaluated by determining its viscoelastic properties [32]. Figure 1 and Figure 2 show the values of the viscoelastic moduli developed, indicating that the use of hydrocolloids affected the viscoelastic properties of CC (*p* < 0.05). In particular, the viscoelastic properties of CC were determined by the properties of the predominant component that formed the protein network [33]. Furthermore, in Figure 2 it can be seen that, in the CC samples containing hydrocolloids (regardless of the type used and concentration applied), the elastic component dominated over the viscous component (*G*′ > *G*″) in the whole frequency region. Thus, it can be stated that, in most of the cases investigated, the model CC samples exhibited a more elastic-like behavior (*G*′ > *G*″); thus, the developed structure of the examined CC samples came “closer” to the ideally elastic behavior. Moreover, the statement above could also be verified by the results depicted in Figure 2. Therefore, increasing the hydrocolloid concentration gave rise to a more elastic CC structure (compared to the CS), which could be attributed to the presence of more intensive interactions and the formation of a denser structure [34].

In Figure 2, a certain trend can be seen where, due to the increase in hydrocolloid concentration, the *G*′ and *G*″ curves increased compared with those of CS. Winter and Chambon [20] reported that, in the case of increasing *G*′, *G*″, and G*, the analyzed products showed higher values of rigidity. The highest values of *G*′ and *G*″ were reported for the CC sample with the addition of κ-carrageenan. Furthermore, the explanation for κ-carrageenan increasing the rigidity of the samples the most is as follows. In general, carrageenans possess the important property of forming complexes with caseins, resulting in the formation of a three-dimensional network (or gel). Casein micelles consist of major fractions of α_S1_-, α_S2_-, β-, and κ-caseins. The reactions of κ-carrageenan and ι-carrageenan with casein micelles are mostly caused by the electrostatic interactions of negatively charged carrageenan sulfate groups with the positively charged region between the 97th and 112th amino acids of κ-casein, resulting in their adsorption onto the micelle’s surface and resulting in the formation of a protective layer [35]. The interactions taking place between KC and casein micelles depend primarily on the system temperature or whether the temperature is above or below the helix–helix transition temperature. Additionally, adsorption will occur only if the carrageenans are in the helical form (below the transition temperature). However, KC adsorption onto the casein micelle is reversible when the system is heated to 60 °C and above. On the contrary, the adsorption of ι-carrageenan is irreversible and no phase separation will occur [36].

In Table 3, the values of *A_F_*, *z*, *G*′, *G*″, G*, and *tan δ* (1 Hz was used as the reference frequency) are presented. From the data obtained, it can be stated that, due to the increasing concentration of hydrocolloids (regardless of the type applied), the values of *A_F_* increased (*p* < 0.05), resulting in an increase in the gel strength of the examined CC samples [37]. The viscoelastic properties of the developed CC samples could also be evaluated using G* values, where increasing values of G* resulted in an increase in the rigidity of the samples [38]. Moreover, from the data presented in Table 3, a clear indication could be obtained that, compared with CS, the CC model samples containing hydrocolloids exhibited higher G* values and the G* values increased with increasing concentration (*p* < 0.05). In addition, KC exhibited the highest G* values, resulting in the highest product rigidity, which could be attributed to more intensive interactions, thus confirming the findings presented in the study by Langendorff et al. [39]. After KC, F samples followed as the samples with relatively higher values of G*, but only at a concentration of 1.00 % (*w*/*w*; Table 3; *p* < 0.05). For the samples with lower concentrations of F, no such significant change in G* values was observed (*p* ≥ 0.05); therefore, it can be stated that a minimum (limiting) concentration of F is probably required to ensure some change in the viscoelastic properties of the product. However, Nagyová et al. [40] or Černíková et al. [34] claimed that the value of the limiting concentration depends on several factors, such as the gel strength of the protein network, the degree of hydrolysis of the proteins present, the pH, or the ionic environment. In the case of the comparison between KC and IK, it can be reported that the KC samples showed relatively higher values of rigidity compared with the IK samples (*p* < 0.05). The presence of a sulfate group that provides a denser structure, may be the explanation for this. In KC, carabiose contains one sulfate group forming rigid gels, whereas, in IK, there are two sulfate groups that form soft elastic gels [35]. The lowest values of G* were reported in the CC sample with the addition of AS, where the values of G* were slightly higher than those of the CS (*p* < 0.05). The reason for this result could be electrostatic interactions with proteins, as sodium alginate forms soluble complexes with β-lactoglobulin at pH 5 [41].

Moreover, from the data in Table 3, the magnitude of the *tan δ*, indicating the degree of viscoelasticity of the CC samples, could be reported. Piska et al. [42] reported that a material with *tan δ* = 1 would behave as a solid and a liquid to the same extent. If *tan δ* < 1, the material would have a more elastic character, whereas if the material has *tan δ* values of > 1, it would present a more viscous character [42]. The *tan δ* values for the CC samples (Table 3) were lower than 1 (at a reference frequency of 1 Hz); thus, the CC samples exhibited more elastic-like behavior. In addition, the values mentioned above (Table 3) were supplemented by the gel strength (*A_F_*) values and interaction factor (*z*) values. On the whole, the statement that there was a change in the CC viscoelastic properties with increasing hydrocolloid concentration (regardless of the type applied) was also confirmed (*p* < 0.05). In this case, there was a significant increase in *A_F_* and a concomitant increase in the *z* values (*p* < 0.05). The latter results are indicative of the number of interacting structural units in the observed polysaccharide–protein network. The increase in *A_F_* values could be mainly attributed to the increasing number of interactions in the developed network structure [43].

### 3.3. Texture Profile Analysis and Spreadability of the Cream Cheese Samples

**Figure 3:**
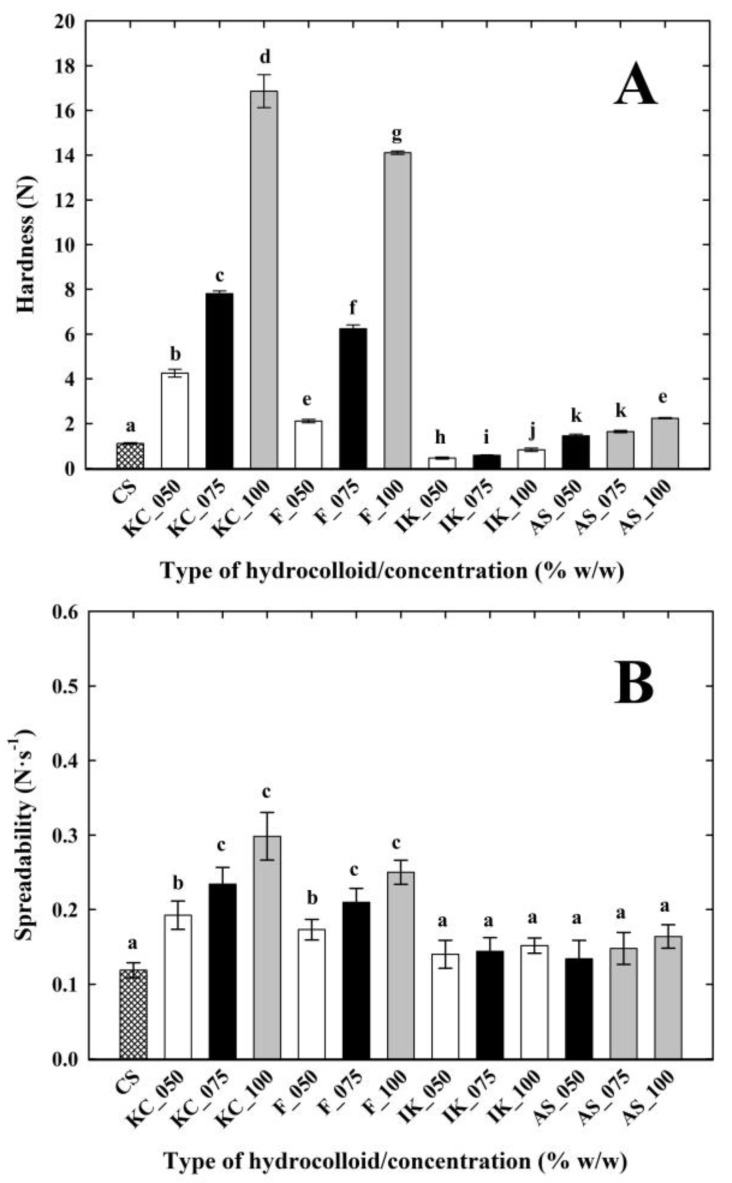
Effect of hydrocolloid type and concentration on the development of the hardness (N; Part (**A**)) and spreadability (N·s^−1^; Part (**B**)) values of the model cream cheese samples manufactured with κ-carrageenan (KC), furcellaran (F), ι-carrageenan (IK), and sodium alginate (AS) at concentrations of 0.50 % (white columns), 0.75 % (black columns), and 1.00 % (gray columns) (*w*/*w*) after 7 days of storage (6 ± 2 °C). The control sample (CS) was also evaluated. Different letters (a–k) indicate significant differences at *p* < 0.05; error bars represent the standard deviation (*n* = 9).

In this study, two approaches were introduced to evaluate the consistency of the CC. One was the rheological analysis mentioned above, which provided information about the samples’ properties under small shear deformations, and the other approach was texture profile analysis (TPA), which described the behavior of the sample under large deformations. Figure 3A shows the evolution of the hardness of the CC samples manufactured with different types of hydrocolloids. In general, the addition of hydrocolloids significantly affected the hardness of the developed CC samples (*p* < 0.05). The results showed that the hardness of all of the CC samples increased with increasing hydrocolloid concentration (regardless of the type utilized) (*p* < 0.05). Moreover, it is also evident (Figure 3A) that the hardness development of all CC samples was gradual with increasing hydrocolloid concentration, while for the samples with KC and F, a rapid increase in hardness was observed at a concentration of 1.00% (*p* < 0.05). This trend could be explained both on the basis of the interaction modes of the tested hydrocolloids and also because, at higher concentrations, interactions occur mainly between the hydrocolloid molecules themselves, leading to higher values of hardness [44]. The highest hardness values were reported for the CC sample with the addition of KC (Figure 3A; *p* < 0.05). Additionally, similar trends have been previously reported by Černíková et al. [34]. Thus, as already mentioned for the results of the rheological analysis, the explanation could stand on the more intensive interactions between the carrageenan chains, resulting in the formation of a “denser” network structure. In general, carrageenan molecules can interact with the protein matrix, leading to an increase in hardness [45]. Other interactions may include hydrogen bonds, hydrophobic, or covalent bonds, which can stabilize the developed protein–polysaccharide matrix [46]. The presence of hydrocolloids would also provide water retention and water absorption capacity in the product, resulting in the formation of stronger gels [30]. On the other hand, low hardness values, similar to the CS values, were observed for CC samples with the addition of IK and AS (Figure 3A). According to Aguilera and Stanley [47], even high concentrations of AS can cause a slight impairment in the compactness of the casein matrix, which would negatively affect the textural properties of the product. The CC sample that presented the lowest hardness values was that with the addition of IK (*p* < 0.05), which confirmed the fact that this hydrocolloid probably formed flexible, but cohesively soft, gels [48].

In Figure 3B, the spreadability values of the CC samples are depicted. The spreadability values were somewhat similar between the CC samples. Furthermore, a certain trend can be seen in which the spreadability value increased with increasing hydrocolloid concentration (regardless of the type applied; *p* < 0.05). The highest values were reported for the CC sample with KC (when comparing the same concentrations of hydrocolloids; *p* < 0.05). The spreadability values of the F samples were higher compared with CS, whereas the resulting IK and AS values were similar to those of CS (*p* < 0.05).

Furthermore, Table 4 shows the values of adhesiveness, stickiness, cohesiveness, and gumminess. The obtained values of adhesiveness (given by the rate of return of the deformed material to its original shape) and stickiness did not differ statistically between the CC samples tested (*p* ≥ 0.05). Moreover, as for the other textural parameters examined, some differences occurred. In particular, not only did the hardness values increase, but also the cohesiveness values increased due to the addition of hydrocolloids (*p* < 0.05). Cohesiveness could be described as the strength of the internal bonds that form a certain food matrix (expressing the force required to remove the adhering substance to a mate) [43,49]. On the contrary, for the samples with F addition, a decreasing trend with increasing hydrocolloid concentration was recorded (*p* < 0.05). The explanation could be found in the interactions of the carrageenan molecule with the protein matrix, leading to both increased hardness and increased cohesiveness [45]. In the case of the gumminess value evaluation, an influence of the addition of hydrocolloids was observed (regardless of the type and concentration used) (*p* < 0.05). In general, the values of gumminess increased with the increasing concentration of hydrocolloids applied (*p* < 0.05). Gumminess expresses the energy required to disintegrate a semi-solid food until it can be swallowed [43]. The lowest gumminess values were reported for the IK samples at concentration levels of 0.50% *w*/*w* and 0.75% *w*/*w* (*p* < 0.05). On the contrary, the highest gumminess values were recorded for the KC sample, in which the gumminess values were further enhanced by the higher addition of hydrocolloids.

### 3.4. Instrumental Color and Emulsion Stability of the Cream Cheese Samples

Color and flavor are very important sensory attributes of dairy products that can influence consumer preferences and food identification [12]. The instrumental color analysis values of CC are shown in Table 5. In general, the addition of hydrocolloids affected most of the CC color parameters (*p* < 0.05). The resulting values showed that all samples could be described as CC of a light-yellow color with a weak green tint. The weakest green tint was reported for the F sample with a concentration of 1.00%. The L* values (Table 5) of all CC samples were close to the value of 100; however, lower L* values were reported for the CC samples with the addition of F or IK. Furthermore, all CC samples exhibited yellow tones; therefore, the h* values were approximately 90°. The C* values (color saturation) were influenced by the hydrocolloid type applied (*p* < 0.05). Moreover, two colors could be distinguished by the human eye depending on their total color difference. Additionally, if *∆E12* < 1, the color differences could not be perceptible to the human eye. In particular, when 1 < *∆E12* < 3, minor color differences could be perceptible to the human eye, and if *∆E12* < 3, the color differences could be perceptible to the human eye. On the basis of our results, we can state that, in most of the CC samples, the color differences would not be perceptible to the human eye [50,51]. Moreover, the addition of hydrocolloids (type or concentration) did not significantly affect the WI of the CC samples tested (*p* ≥ 0.05). 

**Figure 4:**
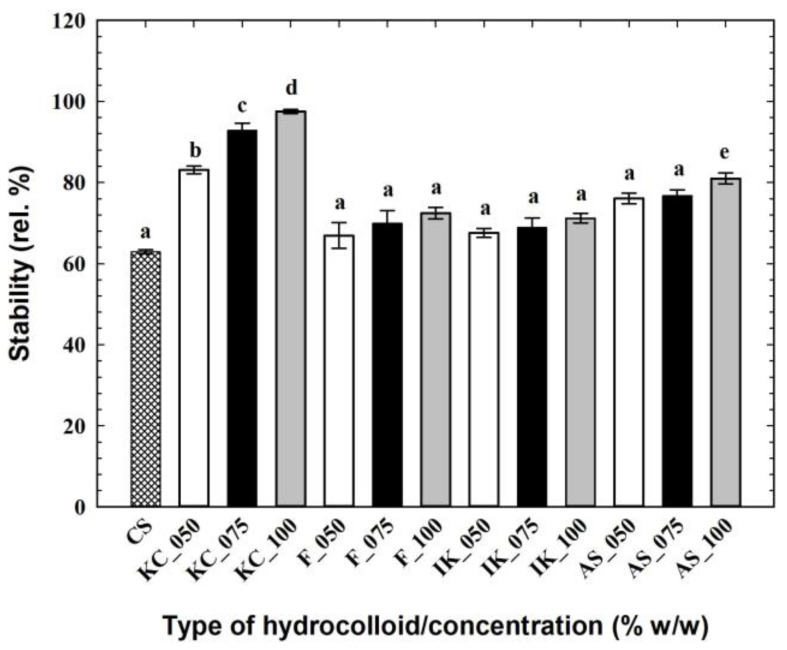
Effect of hydrocolloid type and concentration on the emulsion stability (percentage, rel. %) of the model cream cheese samples with κ-carrageenan (KC), furcellaran (F), ι-carrageenan (IK), and sodium alginate (AS) at concentrations of 0.50 % (white columns), 0.75 % (black columns), and 1.00% (gray columns) (*w*/*w*) and compared with the control sample (CS) after 7 d of storage at 6 ± 2 °C. Different letters (a–e) indicate significant differences at *p* < 0.05; error bars represent the standard deviation (*n* = 9).

The results of CC emulsion stability are shown in Figure 4. From the results obtained, a strong dependence on the hydrocolloid concentration can be seen, where the improvement in sample stability with increasing hydrocolloid concertation was mainly observed for CC samples with KC addition (*p* < 0.05). Furthermore, an increasing trend in the stability values can be observed for all tested samples, as affected by increasing hydrocolloid concentration. The increasing stability of CC may be due to the high affinity of hydrocolloids for water molecules. The reason for this binding is mainly based on the large number of hydroxyl groups [8]. The most stable CC samples were those with the addition of KC (regardless of the concentration used). For the KC samples, the stability was almost 100% and no syneresis phenomenon (water release) was reported. The latter observation could be explained by the fact that KC contains one sulfate group in the galactose unit and, because of the 3,6-anhydride-D-galactose, KC is able to form resistant thermoreversible gel structures. On the contrary, the IK, F, and AS samples exhibited lower emulsion stability values. Hence, it could be reported that KC could be characterized as a more effective thickening agent in CC and similar products due to the amount of K^+^, which is relatively comparable to that of Ca^2+^ (K^+^:Ca^2+^ ≈ 0.82–0.84). The gel-forming ability of both tested carrageenans was influenced by the cations that were capable of neutralizing the sulfate groups with a negative charge. IK was more affected by the presence of Ca^2+^, whereas KC was more affected by the presence of K^+^ ions [37].

### 3.5. Sensory Analysis

The developed model CC samples were subjected to sensory analysis, and the results are depicted in Table 6. All CC samples were positively rated in terms of appearance and color. The best-rated samples in terms of consistency were KC and F with an added concentration of 0.50% (*w*/*w*), which showed excellent consistency. On the other hand, the samples with added IK at concentrations of 0.50% and 0.75% (*w*/*w*) were evaluated as the worst. Moreover, the hardness of CC was also evaluated. and it was found that the hardness of CC increased with increasing concentration of all hydrocolloids used. Assessors rated the KC sample with the addition of 1.00% (*w*/*w*) as the hardest sample, followed by the F sample also with a 1.00% (*w*/*w*) concentration (*p* < 0.05). The results were positively correlated with the rheological analysis or the evolution of the G*, *A_F_*, and *z* values, and similar results were also obtained by Míšková et al. [37]. In the case of spreadability, almost all CCs were rated as products with optimal spreadability, except for the IK samples at concentrations of 0.50% and 0.75% (*w*/*w*). For these samples, the spreadability was the worst and the samples showed a more liquid-like character (*p* < 0.05). Another feature evaluated was flavor. The CC sample with excellent flavor was identified as sample F with a concentration of 1.00% (*w*/*w*). Furthermore, the CC with a very good flavor was sample AS at 0.75% (*w*/*w*), which showed a slight deviation from the excellent flavor. The assessors considered the IK CC samples with concentrations of 0.50% and 0.75% (*w/w*) unacceptable.

## 4. Conclusions

On the whole, it was found that the greatest effect on the viscoelastic and textural properties of the CC samples was reported when KC was used. At the same time, increasing concentrations of the hydrocolloids tested increased the viscoelastic moduli and hardness values of the CC samples. Additionally, the monitored G* and *A_F_* values were higher in the CC samples with KC compared with the products to which F, IK, or AS were added. The resulting values obtained by instrumental color analysis showed that all samples could be characterized as CC of a light-yellow color with a weak green tint. The L* values of all examined CC samples were close to the value of 100, whereas lower values of L* were reported for the samples with added F or IK. Furthermore, from the results of CC emulsion stability, a strong dependence on the concentration of the hydrocolloid applied was reported, where increases in sample stability with increasing hydrocolloid concertation were observed for all CC samples. Therefore, a practical conclusion of the current study can be stated as follows: Consumers who require CC products with softer consistency could be recommended those with a KC concentration of 0.50–0.75% (*w*/*w*) or the use of F or AS (only at concentrations of 1.00% *w*/*w*). On the other hand, in the case where consumers prefer CC products with a more rigid consistency, KC can be used at a concentration of 0.75% or 1.00% (*w*/*w*).

## Figures and Tables

**Table 1:** Raw material formulation and processing parameters of the manufactured cream cheese samples.

Raw Materials and Processing Parameters	Ingredients Composition * (% *w*/*w*)			
	CS	CC1	CC2	CC3
Raw materials				
Quark-type cheese	46.38	45.88	45.63	45.38
Sour cream	39.86	39.86	39.86	39.86
Water	13.04	13.04	13.04	13.04
NaCl	0.72	0.72	0.72	0.72
Hydrocolloid	-	0.50	0.75	1.00
Processing parameters				
Stirring speed (rpm)	3000	3000	3000	3000
Target temperature (°C)	80	80	80	80
Holding time (min) **	10	10	10	10
Total time (min)	16	16	16	16

* CS = Control sample (without hydrocolloid addition); CC1, CC2, and CC3 = Cream cheese samples with hydrocolloid addition. ** Holding time at target temperature.

**Table 2:** Values of dry matter content, pH, and water activity (*a_w_*) of the model cream cheese samples (*n* = 6) **^,1^.

Sample^*^	Hydrocolloid Concentration	Dry Matter	pH	*a_w_*
	(% *w*/*w*)	(% *w*/*w*)	(-)	(-)
CS		29.74 ^a,A^ ± 0.07	4.18 ^a,A^ ± 0.07	0.9982 ^a,A^ ± 0.001
KC	0.50	29.51 ^a,A^ ± 0.05	4.16 ^a,A^ ± 0.08	0.9817 ^a,A^ ± 0.001
	0.75	29.85 ^a,A^ ± 0.08	4.18 ^a,A^ ± 0.04	0.9819 ^a,A^ ± 0.001
	1.00	30.27 ^a,A^ ± 0.15	4.21 ^a,A^ ± 0.01	0.9829 ^a,A^ ± 0.001
F	0.50	30.66 ^a,A^ ± 0.16	4.21 ^a,A^ ± 0.06	0.9925 ^a,A^ ± 0.002
	0.75	30.01 ^a,A^ ± 0.12	4.22 ^a,A^ ± 0.05	0.9911 ^a,A^ ± 0.003
	1.00	29.64 ^e,A^ ± 0.09	4.23 ^a,A^ ± 0.06	0.9923 ^a,A^ ± 0.001
IK	0.50	29.85 ^a,A^ ± 0.12	4.18 ^a,A^ ± 0.05	0.9938 ^a,A^ ± 0.002
	0.75	29.96 ^a,A^ ± 0.09	4.22 ^a,A^ ± 0.01	0.9919 ^a,A^ ± 0.001
	1.00	30.29 ^a,A^ ± 0.02	4.23 ^a,A^ ± 0.01	0.9942 ^a,A^ ± 0.002
AS	0.50	30.01 ^a,A^ ± 0.08	4.19 ^a,A^ ± 0.02	0.9933 ^a,A^ ± 0.001
	0.75	29.87 ^a,A^ ± 0.04	4.21 ^a,A^ ± 0.01	0.9935 ^a,A^ ± 0.002
	1.00	30.06 ^a,A^ ± 0.04	4.18 ^a,A^ ± 0.07	0.9929 ^a,A^ ± 0.001

^1^ Values are presented as the mean ± SD; * CS: control sample; KC: κ-carrageenan; F: furcellaran; IK: ι-carrageenan; AS: sodium alginate; ** Mean values within a column (difference between hydrocolloid type; comparing the same hydrocolloid concentration; the control CC sample was also evaluated) followed by different superscript letters statistically differ (*p* < 0.05). Mean values within a column (difference between hydrocolloid concentration, comparing the same hydrocolloid type; the control CC sample was also evaluated) followed by different uppercase letters differ (*p* < 0.05).

**Table 3:** Values of the gel strength *A_F_*, interaction factor z, elastic modulus *G*′ (at the reference frequency of 1 Hz), viscous modulus *G*″ (at the reference frequency of 1 Hz), complex modulus G* (at the reference frequency of 1 Hz), and loss tangent *tan δ* (at the reference frequency of 1 Hz) of the model cream cheese samples (*n* = 9) **^,1^.

Sample *	Hydrocolloid Concentration	*A_F_*	*z*	*G*′ (kPa)	*G*″ (kPa)	G* (kPa)	*tan δ* (-)
	(% *w*/*w*)	(Pa·s^1/z^)	(-)				
CS		2261.1 ^a,A^ ± 124.5	5.69 ^a,A^ ± 0.02	21.6 ^a,A^ ± 1.5	6.1 ^a,A^ ± 0.5	22.4 ^a,A^ ± 1.3	0.28 ^a,A^ ± 0.01
KC	0.50	62,002.1 ^b,B^ ± 354.7	7.26 ^b,B^ ± 0.03	658.3 ^b,B^ ± 20.4	131.2 ^b,B^ ± 11.3	671.3 ^b,B^ ± 19.8	0.20 ^b,B^ ± 0.01
	0.75	73,085.8 ^c,C^ ± 245.7	7.91 ^c,C^ ± 0.05	674.6 ^c,B^ ± 35.7	149.2 ^c,B^ ± 18.6	690.9 ^c,B^ ± 21.7	0.22 ^c,C^ ± 0.02
	1.00	194,112.1 ^d,D^ ± 145.3	9.95 ^d,C^ ± 0.04	1972.1 ^d,C^ ± 50.7	437.9 ^d,C^ ± 28.9	2020.1 ^d,C^ ± 68.8	0.22 ^d,C^ ± 0.01
F	0.50	17,575.9 ^e,E^ ± 114.2	8.41 ^e,D^ ± 0.02	164.6 ^e,D^ ± 11.8	36.3 ^e,D^ ± 2.7	168.6 ^e,D^ ± 20.1	0.22 ^e,C^ ± 0.01
	0.75	17,926.6 ^f,F^ ± 247.8	9.44 ^f,C^ ± 0.01	178.9 ^f,D^ ± 12.7	37.3 ^f,D^ ± 3.3	182.7 ^f,D^ ± 15.7	0.21 ^f,D^ ± 0.02
	1.00	71,381.8 ^g,G^ ± 456.7	9.57 ^g,C^ ± 0.04	583.3 ^g,E^ ± 25.9	108.9 ^g,E^ ± 12.7	593.4 ^g,E^ ± 26.4	0.19 ^g,E^ ± 0.01
IK	0.50	4614.7 ^h,H^ ± 85.8	6.57 ^h,E^ ± 0.03	45.5 ^h^,^F^ ± 2.6	10.8 ^h,F^ ± 0.9	46.7 ^h,F^ ± 5.1	0.24 ^h,F^ ± 0.01
	0.75	20,357.2 ^I,I^ ± 114.6	6.96 ^i,E^ ± 0.02	151.5 ^i^,^D^ ± 16.4	37.2 ^i,D^ ± 2.2	155.9 ^i,D^ ± 2.8	0.25 ^i,H^ ± 0.01
	1.00	28,772.5 ^j,J^ ± 158.8	7.52 ^j,B^ ± 0.03	282.6 ^j,F^ ± 21.3	64.6 ^j,G^ ± 6.4	289.9 ^j,G^ ± 8.7	0.23 ^j,I^ ± 0.02
AS	0.50	3258.2 ^k,K^ ± 256.7	6.50 ^k,E^ ± 0.01	31.4 ^k,G^ ± 5.1	8.8 ^k,H^ ± 0.9	32.60 ^k,H^ ± 6.7	0.28 ^k,A^ ± 0.02
	0.75	3769.4 ^l,L^ ± 54.7	6.25 ^l,E^ ± 0.04	64.7 ^l,H^ ± 6.4	16.9 ^l,I^ ± 1.3	66.9 ^l,I^ ± 3.7	0.26 ^l,J^ ± 0.02
	1.00	6661.7 ^m,M^ ± 147.6	6.73 ^m,E^ ± 0.05	86.4 ^m,I^ ± 4.7	83.7 ^m,J^ ± 14.5	120.2 ^m,J^ ± 11.8	0.97 ^m,K^ ± 0.01

^1^ Values are presented as the mean ± SD; * CS: control sample; KC: κ-carrageenan; F: furcellaran; IK: ι-carrageenan; AS: sodium alginate; ** Mean values within a column (the difference between hydrocolloid type, comparing the same hydrocolloid concentration; the control CC sample was also evaluated) followed by different superscript letters statistically differ (*p* < 0.05); the samples manufactured using a different hydrocolloid concentration were evaluated independently. Mean values within a column (the difference between hydrocolloid concentration, comparing the same hydrocolloid type; the control CC sample was also evaluated) followed by different uppercase letters differ (*p* < 0.05); the samples manufactured using different hydrocolloid types were evaluated independently.

**Table 4:** Values of adhesiveness, stickiness, cohesiveness, and gumminess of the model cream cheese samples (*n* = 9) **^,1^.

Sample *	Hydrocolloid Concentration	Adhesiveness	Stickiness	Cohesiveness	Gumminess
	(% *w*/*w*)	(N·s)	(N)	(-)	(N)
CS		−0.01 ^a,A^ ± 0.01	−0.04 ^a,A^ ± 0.01	2.83 ^a,A^ ± 0.01	3.14 ^a,A^ ± 0.02
KC	0.50	−0.01 ^a,A^ ± 0.01	−0.01 ^a,A^ ± 0.01	3.06 ^b,B^ ± 0.01	13.01 ^b,B^ ± 0.01
	0.75	−0.01 ^a,A^ ± 0.01	−0.01 ^a,A^ ± 0.01	3.09 ^c,B^ ± 0.01	24.11 ^c,C^ ± 0.01
	1.00	−0.01 ^a,A^ ± 0.01	−0.01 ^a,A^ ± 0.01	3.52 ^d,B^ ± 0.01	59.25 ^d,D^ ± 0.03
F	0.50	−0.03 ^a,A^ ± 0.01	−0.01 ^a,A^ ± 0.01	3.32 ^e,C^ ± 0.01	7.01 ^e,E^ ± 0.01
	0.75	−0.03 ^a,A^ ± 0.01	−0.01 ^a,A^ ± 0.01	3.01 ^f,B^ ± 0.01	18.76 ^f,F^ ± 0.02
	1.00	−0.01 ^a,A^ ± 0.01	−0.01 ^a,A^ ± 0.01	3.15 ^g,B^ ± 0.01	44.45 ^g,G^ ± 0.01
IK	0.50	−0.01 ^a,A^ ± 0.01	−0.03 ^a,A^ ± 0.01	3.17 ^h,B^ ± 0.01	1.45 ^h,H^ ± 0.01
	0.75	−0.02 ^a,A^ ± 0.01	−0.03 ^a,A^ ± 0.01	3.29 ^i,C^ ± 0.01	1.93 ^i,I^ ± 0.01
	1.00	−0.03 ^a^,^A^ ± 0.01	−0.03 ^a,A^ ± 0.01	3.49 ^j,C^ ± 0.01	2.87 ^j,J^ ± 0.02
AS	0.50	−0.02 ^a,A^ ± 0.01	−0.04 ^a,A^ ± 0.01	3.01 ^k,B^ ± 0.01	4.37 ^k,K^ ± 0.01
	0.75	−0.01 ^a,A^ ± 0.01	−0.04 ^a,A^ ± 0.01	3.53 ^l,C^ ± 0.01	5.78 ^l,L^ ± 0.01
	1.00	−0.04 ^a,A^ ± 0.01	−0.04 ^a,A^ ± 0.01	3.20 ^m,C^ ± 0.01	47.17 ^m,M^ ± 0.03

^1^ Values are presented as the mean ± SD; * CS: control sample; KC: κ-carrageenan; F: furcellaran; IK: ι-carrageenan; AS: sodium alginate; ** Mean values within a column (the difference between hydrocolloid types, comparing the same hydrocolloid concentration; the control CC sample was also evaluated) followed by different superscript letters statistically differ (*p* < 0.05); the samples manufactured using different hydrocolloid concentrations were evaluated independently. Mean values within a column (the difference between hydrocolloid concentrations, comparing the same hydrocolloid type; the control CC sample was also evaluated) followed by different uppercase letters differ (*p* < 0.05); the samples manufactured using different hydrocolloid types were evaluated independently.

**Table 5:** Values of lightness (L*), chromaticity on a green-to-red axis (a*), chromaticity on a blue-to-yellow axis (b*), chroma, hue angle (h °), total color difference (*ΔE12*), and whiteness index (*WI*) of the model cream cheese samples (*n* = 9) **^,1^.

Sample *	Hydrocolloid Concentration	L*	a*	b*	C*	*h* (°)	*ΔΕ12*	*WI*
	(% *w*/*w*)							
CS		92.27 ^a,A^ ± 0.12	−0.61 ^a,A^ ± 0.01	14.39 ^a,A^ ± 0.02	14.41 ^a,A^ ± 0.05	92.40 ^a,A^ ± 0.23	-	83.66 ^a,A^ ± 0.01
KC	0.50	92.63 ^b,B^ ± 0.11	−0.61 ^b,A^ ± 0.01	13.94 ^b,B^ ± 0.03	13.96 ^b,B^ ± 0.02	92.50 ^a,A^ ± 0.24	0.57 ^a,A^ ± 0.01	83.22 ^a,A^ ± 0.02
	0.75	92.67 ^c,B^ ± 0.06	−0.45 ^c,B^ ± 0.02	13.73 ^c,B^ ± 0.02	13.74 ^c,B^ ± 0.05	91.90 ^a,A^ ± 0.31	0.79 ^b,B^ ± 0.02	83.43 ^a,A^ ± 0.03
	1.00	92.24 ^d,A^ ± 0.03	−0.53 ^d,C^ ± 0.01	13.90 ^d,B^ ± 0.04	13.91 ^d,B^ ± 0.04	92.20 ^a,A^ ± 0.19	0.49^c,C^ ± 0.01	84.07 ^a,A^ ± 0.01
F	0.50	91.96 ^e,C^ ± 0.02	−0.35 ^e,D^ ± 0.01	14.21 ^e,A^ ± 0.04	14.22 ^e,A^ ± 0.03	91.42 ^a,A^ ± 0.21	0.43 ^d,D^ ± 0.01	83.67 ^a,A^ ± 0.01
	0.75	91.70 ^f,C^ ± 0.14	−0.21 ^f,E^ ± 0.02	14.06 ^f,A^ ± 0.04	14.06 ^f,A^ ± 0.06	90.86 ^a,A^ ± 0.07	0.77 ^e,B^ ± 0.01	83.67 ^a,A^ ± 0.01
	1.00	91.25 ^g,D^ ± 0.08	−0.15 ^g,F^ ± 0.01	14.00 ^g,A^ ± 0.03	14.00 ^g,A^ ± 0.08	90.63 ^a,A^ ± 0.13	1.18 ^f,E^ ± 0.01	83.49 ^a,A^ ± 0.02
IK	0.50	91.49 ^h,E^ ± 0.04	−0.52 ^h,G^ ± 0.01	15.16 ^h,C^ ± 0.01	15.17 ^h,C^ ± 0.04	91.97 ^a,A^ ± 0.24	1.10 ^g,F^ ± 0.02	83.61 ^a,A^ ± 0.02
	0.75	91.81 ^i,C^ ± 0.02	−0.47 ^i,B^ ± 0.01	14.06 ^i,A^ ± 0.03	14.07 ^i,A^ ± 0.03	91.80 ^a,A^ ± 0.18	0.58 ^h,A^ ± 0.02	83.72 ^a,A^ ± 0.01
	1.00	91.71 ^j,C^ ± 0.05	−0.35 ^j,D^ ± 0.01	13.88 ^j,B^ ± 0.01	13.89 ^j,B^ ± 0.04	91.46 ^a,A^ ± 0.05	0.79 ^i,B^ ± 0.01	83.83 ^a,A^ ± 0.01
AS	0.50	92.17 ^k,A^ ± 0.03	−0.63 ^k,A^ ± 0.02	14.82 ^k,A^ ± 0.02	14.83 ^k,A^ ± 0.05	92.45 ^a,A^ ± 0.15	0.44 ^j,C^ ± 0.01	83.23 ^a,A^ ± 0.02
	0.75	92.27 ^l,A^ ± 0.07	−0.59 ^l,A^ ± 0.02	14.45 ^l,A^ ± 0.02	14.46 ^l,A^ ± 0.06	92.35 ^a,A^ ± 0.05	0.06 ^k,G^ ± 0.02	83.61 ^a,A^ ± 0.01
	1.00	91.97 ^m,C^ ± 0.04	−0.63 ^m,A^ ± 0.01	14.79 ^m,A^ ± 0.02	14.80 ^m,A^ ± 0.05	92.45 ^a,a^ ± 0.02	0.50 ^l,C^ ± 0.02	83.16 ^a,A^ ± 0.02

^1^ Values are presented as the mean ± SD; * CS: control sample; KC: κ-carrageenan; F: furcellaran; IK: ι-carrageenan; AS: sodium alginate; ** Mean values within a column (the difference between hydrocolloid types, comparing the same hydrocolloid concentration; the control CC sample was also evaluated) followed by different superscript letters statistically differ (*p* < 0.05); the samples manufactured using different hydrocolloid concentrations were evaluated independently. Mean values within a column (the difference between hydrocolloid concentrations, comparing the same hydrocolloid type; the control CC sample was also evaluated) followed by different uppercase letters differ (*p* < 0.05); the samples manufactured using different hydrocolloid types were evaluated independently.

**Table 6:** Results of the sensory analysis of the CC samples produced. The values are expressed as the median **.

Sample *	Hydrocolloid Concentration (% *w*/*w*)	Appearance	Consistency	Hardness	Spreadability	Flavor	Off-Flavor
CS		1 ^a,A^	1 ^a,A^	3 ^a,A^	4 ^a,A^	1 ^a,A^	1 ^a,A^
KC	0.50	1 ^a,A^	2 ^b,B^	4 ^b,B^	3 ^b,B^	1 ^a,A^	1 ^a,A^
	0.75	1 ^a,A^	3 ^c,C^	4 ^c,B^	4 ^c,A^	1 ^a,A^	1 ^a,A^
	1.00	1 ^a,A^	4 ^d,D^	6 ^d,C^	4 ^d,A^	1 ^a,A^	1 ^a,A^
F	0.50	1 ^a,A^	2 ^e,B^	3 ^e,A^	3 ^e,B^	1 ^a,A^	1 ^a,A^
	0.75	1 ^a,A^	3 ^f,C^	3 ^f,A^	3 ^f,B^	1 ^a,A^	1 ^a,A^
	1.00	1 ^a,A^	3 ^g,C^	4 ^g,B^	4 ^g,A^	1 ^a,A^	1 ^a,A^
IK	0.50	1 ^a,A^	5 ^h,E^	2 ^h,D^	6 ^h,C^	1 ^a,A^	1 ^a,A^
	0.75	1 ^a,A^	5 ^i,E^	2 ^i,D^	6 ^i,C^	1 ^a,A^	1 ^a,A^
	1.00	1 ^a,A^	5 ^j,E^	2 ^j,D^	6 ^j,C^	1 ^a,A^	1 ^a,A^
AS	0.50	1 ^a,A^	6 ^k,F^	6 ^k,E^	6 ^k,C^	1 ^a,A^	1 ^a,A^
	0.75	1 ^a,A^	6 ^l,F^	5 ^l,F^	6 ^l,C^	1 ^a,A^	1 ^a,A^
	1.00	1 ^a,A^	6 ^m,F^	4 ^m,B^	6 ^m,C^	1 ^a,A^	1 ^a,A^

* CS: control sample; KC: κ-carrageenan; F: furcellaran; IK: ι-carrageenan; AS: sodium alginate; ** Median values within a column (the difference between hydrocolloid types, comparing the same hydrocolloid concentrations; the control CC sample was also evaluated) followed by different superscript letters statistically differ (*p* < 0.05); the samples manufactured using different hydrocolloid concentrations were evaluated independently. Median values within a column (the difference between hydrocolloid concentrations, comparing the same hydrocolloid type; the control CC sample was also evaluated) followed by different uppercase letters differ (*p* < 0.05); the samples manufactured using different hydrocolloid types were evaluated independently.

## Data Availability

The data presented in this study are available on request from the corresponding author.
